# Lumpy skin disease: A newly emerging disease in Southeast Asia

**DOI:** 10.14202/vetworld.2022.2764-2771

**Published:** 2022-12-05

**Authors:** Kanokwan Ratyotha, Suksanti Prakobwong, Supawadee Piratae

**Affiliations:** 1 Faculty of Veterinary Sciences, Mahasarakham University, Maha Sarakham 44000, Thailand; 2 Department of Biology, The Parasitology, Geoinformatics, Environment and Health Science Research Group, Faculty of Science, Udon Thani Rajabhat University, Udon Thani 41000, Thailand; 3 One Health Research Unit, Faculty of Veterinary Sciences, Mahasarakham University, Maha Sarakham 44000, Thailand

**Keywords:** *Capripoxvirus*, distribution, lumpy skin disease, newly emerging disease, Southeast Asia

## Abstract

Lumpy skin disease (LSD) is caused by LSD virus (LSDV). This virus has been classified in the genus *Capripoxvirus*, family Poxviridae which generally affects large ruminants, especially cattle and domestic water buffalo. The first outbreak of LSD was found in 1929 in Zambia, then spreading throughout Africa and with an ongoing expanding distribution to Asia and Europe. In 2020, LSD was found from Southeast Asia in Vietnam and Myanmar before reaching Thailand and Laos in 2021. Therefore, LSD is a newly emerging disease that occurs in Southeast Asia and needs more research about pathology, transmission, diagnosis, distribution, prevention, and control. The results from this review show the nature of LSD, distribution, and epidemic maps which are helpful for further information on the control and prevention of LSD.

## Introduction

Lumpy skin disease (LSD) is caused by LSD virus (LSDV), a virus in the family Poxvirus, genus *Capripoxvirus* as well as sheep pox virus (SPPV) and goat pox virus (GTPV) [1]. This virus can cause infection mainly in cattle (*Bos* spp.) and buffaloes (*Bubalus* spp.); there are also reports in other wild ruminant species, such as giraffes, bulls, and springboks [2]. This arbovirus is probably transmitted by mechanical transmission through blood–sucking arthropods, including mosquitoes, ticks, and flies [3]. This virus can also be transmitted to susceptible animals through direct contact with the secretions of other infected animals and indirect contact from contaminants of the owner of the animals and objects (vehicle, equipment, etc.) [1, 4]. Infected animals may present variations of clinical signs ranging from subclinical to high morbidity and mortality. The clinical signs are fever (40.0°C–41.5°C), lacrimation, nasal discharge, hypersalivation, lethargy, anorexia, and weakness, followed by the development of nodular lesions in the skin and mucous membranes of the whole body. In addition, these lesions may develop into the muscular layer [3, 4]. The resulting wound lesions can develop necrotic tissue and scarring, which may occur with secondary infection with other types of complications such as bacteria, viruses, or myiasis and cause severe clinical symptoms [5]. In general, prevalence of LSD ranges from 1%–2% to 80%–90% in different situations in the endemic region [2]. Mostly LSD can cause low mortality and the differences in the mortality rate may be explained by differing susceptibility of hosts (strain, age, and host immune response).

The first reported case of LSD occurred in 1929 in Zambia and after that, it spread throughout South Africa with sporadic outbreaks in some areas [5]. At present, this disease is an endemic disease in Africa. Lumpy skin disease has recently spread in Asia during 1988–1989 following outbreaks in Europe and the Middle East in 1990 [6]. The disease emerged in South Asia in 2019 and then rapidly spread throughout Southeast Asia in 2020 [1]. Infection with LSDV could affect not only the health status of ruminants, but furthermore, might impact the economic activity of ruminants, especially cattle.

Here, we review the general information about LSD and show where research is needed for a better understanding of the biology, pathology, transmission, diagnosis, distribution, prevention, and control of this newly emerging disease in Southeast Asia.

## Virus and Classification

**Figure-1:**
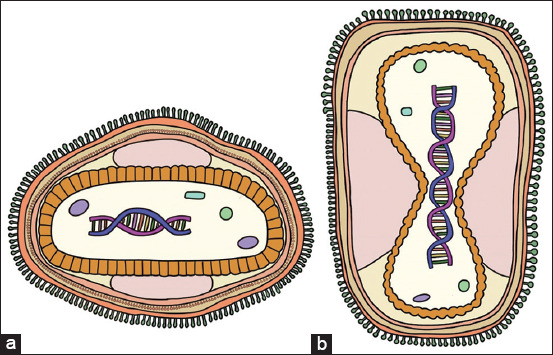
Schematic diagram of the poxvirus structure. (a) cross-section; (b) longitudinal section. (Figure prepared by Kanokwan Ratyotha).

Lumpy skin disease virus (LSDV) is a virus in the family Poxviridae, subfamily Chordopoxviridae, genus *Capripoxvirus*. The genus *Capripoxvirus* comprises three viruses; SPPV, GTPV, and LSDV. Lumpy skin disease virus is large–sized (230–260 nm) enclosed in a lipid enveloped with a genome of approximately 150 kilobase pairs (kbp) and shared 97% identity in the nucleotide sequences with SPPV and GTPV genome (Figure-1). The LSDV genome included at least 146 putative genes, which displayed proteins that play roles in virion structure, DNA replication, transcription and metabolism, protein processing and assembly, virus stability, and evading host immune response [7]. Lumpy skin disease virus can cause infection mainly in large ruminants; specifically in cattle, buffaloes, and other wild ruminant species. However, the disease is not contagious from animals to humans. Signs and symptoms of LSDV vary widely and depend on many factors, such as status of the host’s immune response, strain of the virus, and the environment. Severe clinical signs of disease may occur in young animals, lactating animals, or animals with lower immunity than healthy animals. European beef cattle strains (*Bos taurus*) are more susceptible to LSD than tropical or Indian beef cattle strains (*Bos indicus*). Moreover, domestic buffalo (*Bubalus bubalis*) have a lower incidence rate than cattle [1, 4, 8]. The LSDV genome can be detected in nodules, ulceration, blood, secretions, and semen in both vertebrate (ruminant) (Table-1) [1, 2, 4] and invertebrate animals (arthropods) (Table-2) [9–19].

**Table-1:** Vertebrate hosts susceptible to LSDV infection.

Vertebrate hosts	Countries/Regions	References
Giraffe		
*Giraffa camelopardalis*	South Africa	[1, 4]
Impala		
*Aepyceros melampus*	South Africa	[1, 4]
Eland		
*Taurotragus oryx*	South Africa	[1]
Wildebeest		
*Connochaetes gnou*	South Africa	[1]
Thomson’s Gazelle		
*Eudorcas thomsonii*	South Africa	[1]
Oryx		
*Oryx leucoryx* *Oryx gazelle*	South Africa, Saudi Arabia	[1, 2, 4]
	South Africa, Saudi Arabia	[1, 2]
Springbox		
*Antidorcas marsupialis*	Namibia	[1, 2, 4]
African wild buffalo		
*Syncerus caffer*	Kenya	[1, 2]

LSDV=Lumpy skin disease virus

**Table-2:** LSDV genome detected in vertebrate animals.

Invertebrate hosts	Countries/Regions	References
Ticks		
*Rhipicephalus appendiculatus*	South Africa	[9–12]
*Amblyomma hebraeum*	South Africa	[9–11, 13–15]
*Rhipicephalus decoloratus*	South Africa	[13, 15]
Glimpses		
*Haematopota* spp.	South Africa	[16]
Flies		
*Stomoxys calcitrans*	Belgium, Egypt	[16, 17]
Mosquitoes		
*Aedes aegypti*	Egypt	[18]
*Anopheles stephensi*	Egypt	[17]
*Culex quinquefasciatus*	Egypt	[17]
*Culicoides nubeculosus*	Egypt	[19]

LSDV=Lumpy skin disease virus

## transmission, Spread, and Virus Stability

Lumpy skin disease is a vector-borne disease transmitted by mosquitoes (*Aedes aegypti, Anopheles stephensi, Culex quinquefasciatus*, and *Culicoides nubeculosus*), ticks (*Rhipicephalus appendiculatus, Rhipicephalus decoloratus*, and *Amblyomma hebraeum*), and Diptera (*Haematopota* spp. and *Stomoxys calcitrans*). The LSDV can survive in skin nodules for 1 month and at least 3 weeks in air–dried hides. The virus is excreted in the blood, nasal secretions, saliva, ear notches, semen, and milk and can be transmitted to suckling calves [20–22]. In general, vectors enhance the distribution of LSDV by mechanical and biological transmissions. Many studies have reported that after blood-sucking vectors (mosquitoes, ticks, glimpse, and flies) take a blood meal from infected cattle (viremia stage), the virus can propagate and shed in the salivary glands, head, body, and feces of insects. This allows the infected insects to become a reservoir for further transmission [15]. The infectivity of LSDV has been studied in both egg and juvenile of ticks (*R. decoloratus*) in which it was found that the disease can be transmitted by transovarial transmission [14]. In addition, the mechanical contact of flies is also a way they can be carriers of disease. Direct contact between cattle in a cage has been commonly found in endemic areas. However, the viral transmission was also accomplished by the contamination of veterinary equipment and vehicles as well as stockholders from a farm translocating to distant areas [1, 23]. Most LSD infections have been found in the summer when vectors are active; it may designate the blood–feeding insects and the virus spread. The enhancement of risk factors was associated with a warm and humid climate that supported the reproduction of vector populations. The introduction of new animals to a herd is one of the risk factors.

Lumpy skin disease virus is well tolerated in the environment in the pH range of 6.3–8.3 and it can survive in dry scabs on the skin for up to 3 months [1]. Lumpy skin disease virus can grow in cell cultures at 4°C for up to 6 months, in phosphate buffer saline at 28°C for up to 35 days, in skin nodule lesions collected from frozen at –80°C for up to 10 years. However, it can be destroyed by ultraviolet heat for an exact time, for example, 55°C for 2 h, 60°C for 1 h, and 65°C for 30 min. Moreover, this virus is sensitive to excess acid or base and therefore, it can be destroyed by common disinfectants [9].

## Pathogenesis and Clinical Signs

In some outbreaks that occurred in Africa and middle Asia countries, the mortality rate was generally low (1%–3%) but may reach 40% in some regions. After infection, the incubation period ranges from 4 to 7 days, as determined experimentally, but for naturally occurring infections, the incubation period is 28 days or is prolonged to 35 days [1, 4]. Lumpy skin disease virus infection by intradermal replication in fibroblasts, macrophage, pericytes, and endothelial cells leads to viremia causing vacuities and lymphangitis in affected areas [2]. After cattle recover from infection, they acquire antibodies for about 6 months [6]. Lumpy skin disease is an economically impact disease in an outbreak because the severity in cows peaks during lactation and causes a decreased milk harvest during the high fever caused by the viral infection and bacterial mastitis. Clinical signs after the incubation period can be classified into 4 phases.

Phase 1 (acute phase) after the incubation period, animals have fever as high as 41°C for about 7 days sometimes prolonged to 10 days with anoxia, depression, lacrimation, increased nasal discharge, saliva secretions, lack of milk, found multinodular lesions around skin, and mucous membrane. Some cases are non–febrile.

**Figure-2:**
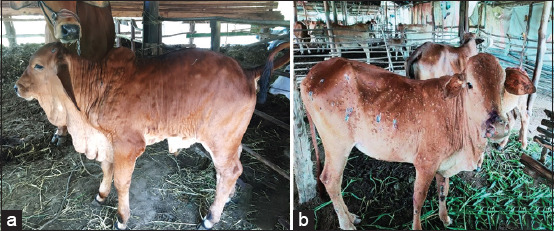
The clinical sign of lumpy skin disease in cattle in Thailand. (a) The lymph nodes develop noticeably enlarged 3–5 times of its normal; (b) the nodule lesions’ transformation into ulceration and necrosis.

Phase 2 subscapular and precrural lymph nodes develop noticeable enlargement 3–5 times of their normal, and there are increased multi nodules, mostly on the head, neck, limbs, genitalia, udder, mucous membrane, nasal and oral cavities, or plaques at the site of inoculation. The diameter of the nodule lesion is 0.5–5 cm, apparent in varying numbers and sizes, from only a few to multiple lesions covering the entire animal (Figure-2a). After 1–2 days, nodules rupture, also shedding virus depending on the concentration of virus. Sometimes found edema of limbs is caused by lymphangitis and vasculitis.

Phase 3, after 2–3 weeks, nodules lesions into ulceration and become necrotic. Also, beaded serum exudes, especially from limbs and causes lameness, pain, and lack of movement. In severe cases, an ulcerative lesion appears in the mucous membranes at various points, such as the eye and nasal cavities; there is excessive salivation, lachrymation, and nasal discharge. The secretions from animals may contain LSDV [19].

Phase 4, after at least 1 month, there is complete healing of ulcerations and also thickening of the skin and hyperpigmentation of the lesion (Figure-2b).

Bacterial and virus infections can occur in lesions, and these pathogens are able to be inhaled by the host, causing a sequence of complications. These complications include keratitis, mastitis, pneumonia, and myiasis and can increase mortality. Other clinical signs of complications are abortion, decreased lactation, anestrus, infertility, and subclinical sign may be present. Post-mortal LSDV infected cattle have been found with nodule formation and ulceration in the trachea, lung, and gallbladder [6, 19, 24]. Differential diagnosis include pseudo LSD/bovine *Herpes* mammillitis by bovine *Herpes* virus type 2, pseudocowpox and bovine papular stomatitis by *Parapoxvirus*, insect biting, urticaria, and demodicosis [4]. In outbreaks of the disease, the morbidity rate varies widely depending on the immune status of the hosts and the abundance of mechanical arthropod vectors.

Lumpy skin disease virus affects large ruminants, especially cattle and domestic water buffalo; however, this virus has also been reported in wildlife due to these animals belonging to the suborder Ruminantia, as well as cattle and buffaloes. Clinical signs of LSDV infection in wildlife are difficult to monitor and may range from asymptomatic to severe clinical signs [24]. Although reports of disease in wildlife are low incidence, it is still a concern whether the disease spreads from pets to wildlife [25]. It is difficult to prevent and control the disease due to the inability to control livestock and make preventive vaccines for wildlife as well as livestock. There are also many species of ruminant wildlife that are not known how to transmit disease, which can affect their natural population.

## Humoral and Cellular Immunity

In cases of natural infection, the immunity caused by infection can be detected approximately 2 weeks after infection. From experimental infection, antibodies are detectable from 6 to 8 day post-infection. The highest immunity can be detected during 3–4 weeks after infection and remain detectable for up to 5 months [26]. Although antibodies are able to limit the spread of extracellular organisms, most LSDV are predominantly intracellular. Humoral immunity cannot be enough to eliminate the proliferation of viruses inside cells. Therefore, cell-mediated immunity is essential for the effective control of infection in animals. Once an animal has been vaccinated, the humoral immune response can last longer than 7 months which is capable of preventing disease [27]. However, animals in endemic regions are recommended to receive an annual vaccination booster due to the duration of humoral and cellular immunity is still unknown.

## Diagnosis

Diagnosis of LSDV is based on characteristic clinical signs combined with various laboratory approaches. Virus isolation and electron microscopy can be done but are rather expensive, labor and time–consuming, and cannot differentiate between poxvirus virions. The immunological–based techniques such as enzyme-linked immunosorbent assay have been developed to detect antibodies for LSDV infection; however, false detection caused by non-specific binding can occur between *Parapoxvirus* and *Capripoxvirus* [28]. In the laboratory, DNA–based detection methods by polymerase chain reaction (PCR) or real-time-PCR (RT-PCR) is used to detect viral DNA in specimens, including nodules, secretion, semen, and blood of suspected animals [29]. Target genes for PCR or RT-PCR detection are often used for the gene–specific viral attachment proteins such as P32, RPO30, and GPCR [30, 31]. For diagnosis, LSDV, sheep and goat poxviruses can be distinguished by real-time PCR technique [32]. The differentiation among natural genotypic targets of either vaccine or field strain genomes was developed by using a universal TaqMan probe to cover the field, vaccine, and recombinant strains of LSD [33]. The virulent LSDV from the vaccine strain was established by the restriction fragment length polymorphism [34].

## Necropsy and Histopathological Finding

Infection with LSDV classically causes an acute disease with fever, depression, and appearance of nodules and lesions in the skin. The clinical signs of LSD are lymph nodes to form a skin blister about 2–5 cm in diameter on the skin, such as head, neck, legs, breast, and genitals. The necrotic nodules were ulcerated and formed deep scabs [35]. Larger vesicles may become necrotic and scarred for several months, while smaller vesicles heal faster. Blisters or ruptures of the cyst can be found. The mucus in the mouth, gastrointestinal tract, trachea, and lungs may be seen with edema. For asymptomatic infection, lesions are found in the subcutaneous or muscular layer. After the postmortem, lesions are often found in the respiratory organs, gastrointestinal tract, breast, lungs, bladder, kidneys, uterus, or testicles [36]. Histopathological examination of nodular skin biopsies showed edema, hyperemia, acanthosis, hydropic degeneration, and hyperkeratosis in epidermis [37].

## Epidemiology (Susceptibility Hosts, Prevalence in Other Countries)

**Figure-3:**
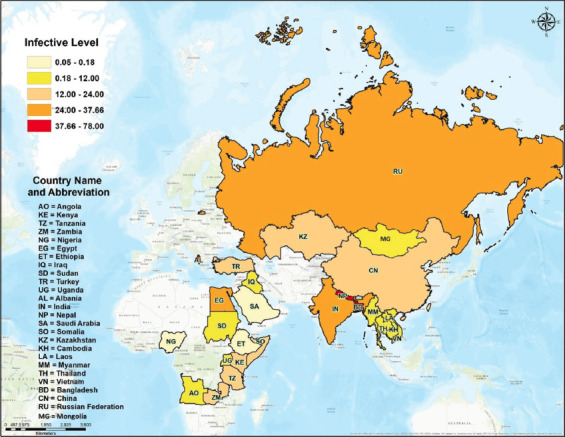
Prevalence of lumpy skin disease positively detected by polymerase chain reaction assay during 2016–2022. [Source: base map from the public Geo-Informatics and Space Technology Development Agency (GISTDA) using ArcGIS software (ESRI Inc., Redlands, CA, USA)].

Lumpy skin disease virus was first investigated in Zambia in 1929 and was endemic in Africa during 1988–1989. However, the disease had been transmitted to middle Asian countries, including Saudi Arabia, Iran, Israel, and Iraq, by 1990 [6]. The incidence of LSD worldwide in 2016–2020 included African and Asian countries, and the epidemiology dynamically changed between the years. The prevalence and incidence of LSD detected by RT-PCR were reviewed during 2016–2020 (Figure-3). In 2016, the prevalence of LSD in the countries was 4.66% in Iraq [38], 4.77% in Uganda [39], 5.00% in Nigeria [40], 6.00% in Saudi Arabia [23], 7.22%–18.0% in Ethiopia [41, 42], and 12.9%–22.00% in Kazakhstan [43, 44]. In 2017, the prevalence of LSD in countries was 24.00% in Egypt [45] and 5.67% in Ethiopia [46]. In 2018, the prevalence of LSD was 29.00% in Russia [47] and 31.20%–88.80% in Egypt [48–50]. In 2019, the prevalence of LSD was 10.00% in Bangladesh [51], 19.50% in China [52], 22.28%–27.50% in Egypt [36, 53], and 37.66% in India [54]. In 2020, the prevalence of LSD was 3.00%–6.00% in Myanmar [55], 4.85% and 53.20% in Nepal [56, 57], 13.93% in India [58], and 78.00% in Bangladesh [59]. In 2021–2022, the prevalence of LSD was 70% in Egypt [60], 4.17% in Thailand [61], 5.9% in Mongolia [62], and 36.2% in Ethiopia [63].

## Prevalence of LSD in Southeast Asia

Lumpy skin disease was shown to be distributed to South Asia through Southeast Asia (SEA) in 2019–2020 [1]. The first detection was found in the upper part of Vietnam in 2020 by the World Organization for Animal Health or Office International des Epizooties; OIE. The virus was isolated, identified, and investigated. The virus was similar to that endemic in Russia in 2017 and in China in 2019, indicating the disease was introduced from China–Vietnam border, and became distributed through 27 provinces in the country [64, 65]. Thereafter, LSD was transmitted to other countries in SEA, such as Laos, Cambodia, Thailand, and Myanmar [55, 66, 67]. In Malaysia, the disease has been reported but some cases have not been confirmed as LSD [68]. In Indonesia and the Philippines, LSD has not been found and critical notification in the prevention and control of LSD were concerned [69, 70]. In Thailand, at least 65 of 76 Provinces had reported infected animals to OIE by April, 2021. The Thai government produced strategies for prevention and controls, such as non–transfer of the animals from endemic areas, and vaccination in cattle and buffalo [71], which resulted in a sharp drop in morbidity rate in cattle in Thailand in 2022. Our experiences are agreeable with the encounter of LSD in Turkey in 2013 where uncontrolled animal movement is an important risk factor for spreading LSD to the neighboring countries, including Balkan, Caucasus, Iran, and Asia. Therefore, when any case occurs, isolation, quarantine, and vector control are necessary and have to be applied immediately [72].

## Prevention Control and Eradication (Risk Factors, Regulations, Actions, and Vaccinations)

The stability of viruses in ambient conditions for a long period has certainly been established. It can persist in desiccated lesions on skin for 25–50 days and persist for many months in the dark environment in animal sheds. Veterinary education is needed for livestock workers to enable the performance of timely diagnoses of the disease to diminish the spread of the disease. Effective treatment against LSD has not been recognized. Symptomatic treatment for anti–inflammatory symptoms and antibiotics for preventing secondary microbial infection was used. Supportive therapy such as the Vitamin B–complex, Vitamin AD3E to retain the feeding capacity, and reproductive maintenance was frequently used (Table-3) [73–75].

**Table-3:** Therapeutic agents for LSD treatment.

Therapeutic agents	Pharmacological effects	References
Enrofloxacin	Antibiotic	[73, 74]
Oxytetracycline		[73, 75]
Penicillin		[75]
Cephalosporin		[75]
Tetracycline		[75]
Fluoroquinolone		[75]
Chlorpheniramine maleate	Antihistamine	[73, 74]
Meloxicam	Nonsteroidal anti–inflammatory	[73, 74]
Dexamethasone suspension	Steroidal anti–inflammatory	[75]

LSD=Lumpy skin disease

Disease spreading can occur by infected animals or contaminated equipment or vectors. Early outbreaks can be controlled if the animal population is quarantined, sanitation on equipment or locality and biosafety. Controlling the movement or quarantine of newly imported animals for at least 3–4 weeks before being imported to the farm is one of the practices during the epidemic period [26]. Blood–sucking insects are the main vectors that cause the rapid spread of the disease; therefore, destroying breeding grounds, removing manure and cleaning with pesticides to disinfect and eliminate vectors regularly are recommended. Moreover, vaccination is an important preventive measure to reduce the spread and severity of the disease.

To prevent and control strategies, including the assortment of risks and affected animals, movement restrictions, and compulsory and consistent vaccination are recommended, including the following:

### Restriction of animal movement

The movement of animals infected with LSDV and/or effects of LSD must be exactingly prohibited to prevent the distribution of the disease to other areas and crossover to non-infected farms. The prevention of transboundary movements and the restriction of animal movement should be strict. Animals with skin lesions should be investigated and should be isolated for assessment.

### Control the distribution of vectors

The distribution of arthropod vectors’ movement and spread is risk factors for LSD transmission. Insecticides and traps are frequently used in livestock to prevent the disease.

### Vaccination

The immunization of LSD by live attenuated vaccines has been effectively used in endemic areas. Antigenic homology of *Carpipoxvirus*, including SPPV, GTPV, and LSDV, cross-protection of immune response was beneficial [1, 4, 24, 76]. A live attenuated vaccine is commercially available for LSD eradication. Sheep pox vaccine from SPPV and GTPV is used for control in countries with high LSD outbreaks. Types of LSD vaccines are as follows: (1) Attenuated LSDV vaccines (Neethling vaccines) are the currently effective vaccine to prevent LSD in cattle. The effective control success possibility is 80% in the livestock, (2) Attenuated SPPV vaccines are suitable for the areas that SPPV and LSDV outbreak and (3) Attenuated Gorgan GTPV vaccine is suitable for the areas where outbreaks are a combination of SPPV and LSDV [1]. The live attenuated LSDV vaccine (Neethling vaccines), which is used in livestock worldwide, is the only ubiquitous LSDV vaccine. After vaccination, the immunity is raised within 10–30 days. This vaccine is recommended at any age unless contemporaries showing signs of infection have already occurred.

## Conclusion

Lumpy skin disease is an infectious disease in large ruminants, cattle, and domestic water buffalo. This disease regularly occurs in Africa, Europe, and some regions of Asia and spread to Southeast Asia in 2020. The clinical signs range from subclinical to high fever, lymph node enlargement, and apparent nodules over the entire body, followed by developing necrotic tissue and scarring. Although LSD has a low mortality rate, the development of lesions can cause complications and has no specific treatment. Successful prevention of LSD is vaccination together with vector control, controlling animals, especially from endemic regions, and monitoring the situations of LSD outbreaks continuously by disease surveillance.

## Authors’ Contributions

KR: Performed literature search and drafted the manuscript. SukP: Reviewed and edited the manuscript. SP: Conceived the study, reviewed and edited the manuscript, and performed final manuscript revision. All authors have read and approved the final manuscript.
